# Characterisation of DNA methylation changes in *EBF3* and *TBC1D16* associated with tumour progression and metastasis in multiple cancer types

**DOI:** 10.1186/s13148-019-0710-5

**Published:** 2019-08-05

**Authors:** Euan J. Rodger, Aniruddha Chatterjee, Peter A. Stockwell, Michael R. Eccles

**Affiliations:** 1 Department of Pathology, Dunedin School of Medicine, University of Otago, 56 Hanover Street, Dunedin, 9054 New Zealand; 2 Maurice Wilkins Centre for Molecular Biodiscovery, Level 2, 3A Symonds Street, Auckland, New Zealand

**Keywords:** DNA methylation, Cancer, Metastasis, Promoter, Gene body

## Abstract

**Background:**

Characteristic DNA methylation differences have been identified between primary and metastatic melanomas at *EBF3* and/or *TBC1D16* gene loci. To further evaluate whether these epigenetic changes may act more generally as drivers of tumour onset and metastasis, we have investigated DNA methylation changes involving *EBF3* and *TBC1D16* in additional publicly available data of multiple different tumour types.

**Results:**

Promoter hypermethylation and gene body hypomethylation of *EBF3* were observed in a number of metastatic tumour types, when compared to normal or primary tumour tissues, as well as in tumour vs normal tissues and in a colorectal primary/metastasis pair, although not all tumour samples or primary/metastasis cancer pairs exhibited altered patterns of *EBF3* methylation. In addition, hypomethylation of *TBC1D16* was observed in multiple tumours, including a breast cancer primary/metastasis pair, and to a lesser degree in melanoma, although again not all tumours or cancer primary/metastasis pairs exhibited altered patterns of methylation.

**Conclusions:**

These findings suggest characteristic DNA methylation changes in *EBF3* and *TBC1D16* are relatively common tumour-associated epigenetic events in multiple tumour types, which is consistent with a potential role as more general drivers of tumour progression.

## Background

Cancer is a leading cause of death worldwide. Over the recent decades, many mutations have been identified that promote tumour growth (i.e. so-called “driver” mutations) [1]. However, evidence for the existence of unique or metastasis-specific genetic mutations that drive cancer metastasis has remained elusive. Although metastatic tumour cells are responsible for at least 90% of cancer-related deaths [2, 3], which is in part due to their ability to spread to distant organs via the lymphatics or circulatory systems, recently, Vogelstein and colleagues have suggested that due to the lack of metastasis-specific mutations being identified from large-scale next-generation sequencing approaches, specific driver gene mutations causing metastasis do not exist [4].

It is now well established that epigenetic changes are associated with tumour growth [5]. Primary tumour cells need to undergo a series of dynamic changes to enable them to successfully metastasise [3, 6]. The plastic nature of the cancer epigenome also makes it highly plausible that epigenetic alterations are a characteristic change that drives cancer cells towards metastasis [7, 8]. However, to date, relatively few studies have investigated whether characteristic epigenetic alterations, that involve one or more loci, are epigenetic drivers (epi-drivers) of metastasis and possess critical roles in advanced stages of tumourigenesis in multiple different cancer types. While a precise framework has yet to be described to identify such epi-driver changes, we recently proposed an analytical framework to identify and assign functional significance to putative epi-drivers of metastasis [7]. We defined epi-drivers to be epigenetic alterations that can definitively be shown to be required for at least one of the cancer hallmarks. An epi-driver change would be heritable in daughter cancer cells, dependent on the cancer hallmark, with generalised epi-drivers hypothesised to occur across multiple cancer types, and tumour-specific epi-drivers confined to just one cancer type. In contrast, epigenetic passengers (epi-passengers) would not be required for a hallmark feature of cancer. We have proposed that there is merit in studying paired primary and metastatic tumours, where there is an opportunity to directly compare epigenetic changes in metastatic lesions with a primary tumour [7]. In this approach inter-patient heterogeneity and to some degree mutational heterogeneity (different mutational signatures in different tumours) could largely be avoided.

Recent genome-wide tumour studies have used comparative analysis of paired primary and metastatic tumour samples to identify epi-drivers of cancer metastasis [9, 10]. Of these studies, Chatterjee et al. [9] observed elevated mRNA levels and promoter hypermethylation of Early B Cell Factor 3 (*EBF3*) in metastatic melanoma cell lines compared to matched primary melanoma cell lines. Subsequent functional analyses revealed this gene has an oncogenic role. Promoter hypermethylation and elevated mRNA levels of *EBF3* were validated in an independent melanoma cohort (data generated by Marzese et al. [11, 12]) and in The Cancer Genome Atlas melanoma dataset (TCGA SKCM), which contains data for 458 patients (99 primary and 359 metastatic tumours) [9]. In another study, Vizoso and colleagues [13] analysed primary and metastatic cell line pairs from melanoma, breast and colorectal cancer to identify common DNA methylation-associated changes involved in the formation of metastasis. Their analysis identified TBC1 domain family member 16 (*TBC1D16*) as a potential epi-driver of metastasis. Loss of *TBC1D16* methylation was associated with activation of an alternative cryptic transcript, *TBC1D16-47KD*, which was shown to promote melanoma proliferation and metastasis. Further in vitro and in vivo functional analyses indicated that TBC1D16-47KD promoted melanoma proliferation and metastasis, possibly by regulating Rab GTPases and EGFR activation. Hypomethylation of *TBC1D16* was also shown to increase sensitivity to BRAF and MEK inhibitors but predicted poorer clinical outcome for melanoma patients [13].

A recent genome-wide study by Wouters et al. [10] identified significant hypermethylation of 5808 Illumina 450k probes (1533 genes) and significant hypomethylation of 4151 probes (1722) in both primary melanomas and metastases compared to benign nevi. In their analysis, seven CpGs in the gene body of *EBF3* (chr10:131636622–131671489, GRCh37; cg03774288, cg07890827, cg09121772, cg09371530, cg09649486, cg16803064 and cg25866634) showed significant loss of methylation in metastases compared to benign nevi, and one of these CpG sites showed the same degree of hypomethylation in metastases compared to primary melanoma. Furthermore, they identified three CpGs in *TBC1D16* that showed a significant decrease in methylation in metastases compared to primary melanoma. Two of these CpG sites were in the dataset originally identified by Vizoso et al. [13] as being hypomethylated in metastases.

In previous genome-wide DNA methylation analysis of three cutaneous primary and metastatic melanoma cell line pairs using reduced representation bisulfite sequencing (RRBS), we showed that an RRBS fragment in the promoter of *EBF3* (at chr10:131763530–131763587, GRCh37) was significantly hypermethylated in metastatic cell lines compared to matched primary cell lines [9]. In the present study, we have investigated DNA methylation changes in *EBF3* and *TBC1D16* in publicly available data of multiple different tumour types, so as to further evaluate the potential role of these two genes associated with tumourigenesis and metastasis.

## Results

### DNA methylation differences in *EBF3* and *TBC1D16* occur between primary melanoma and metastatic melanoma tumour tissues

**Fig. 1:**
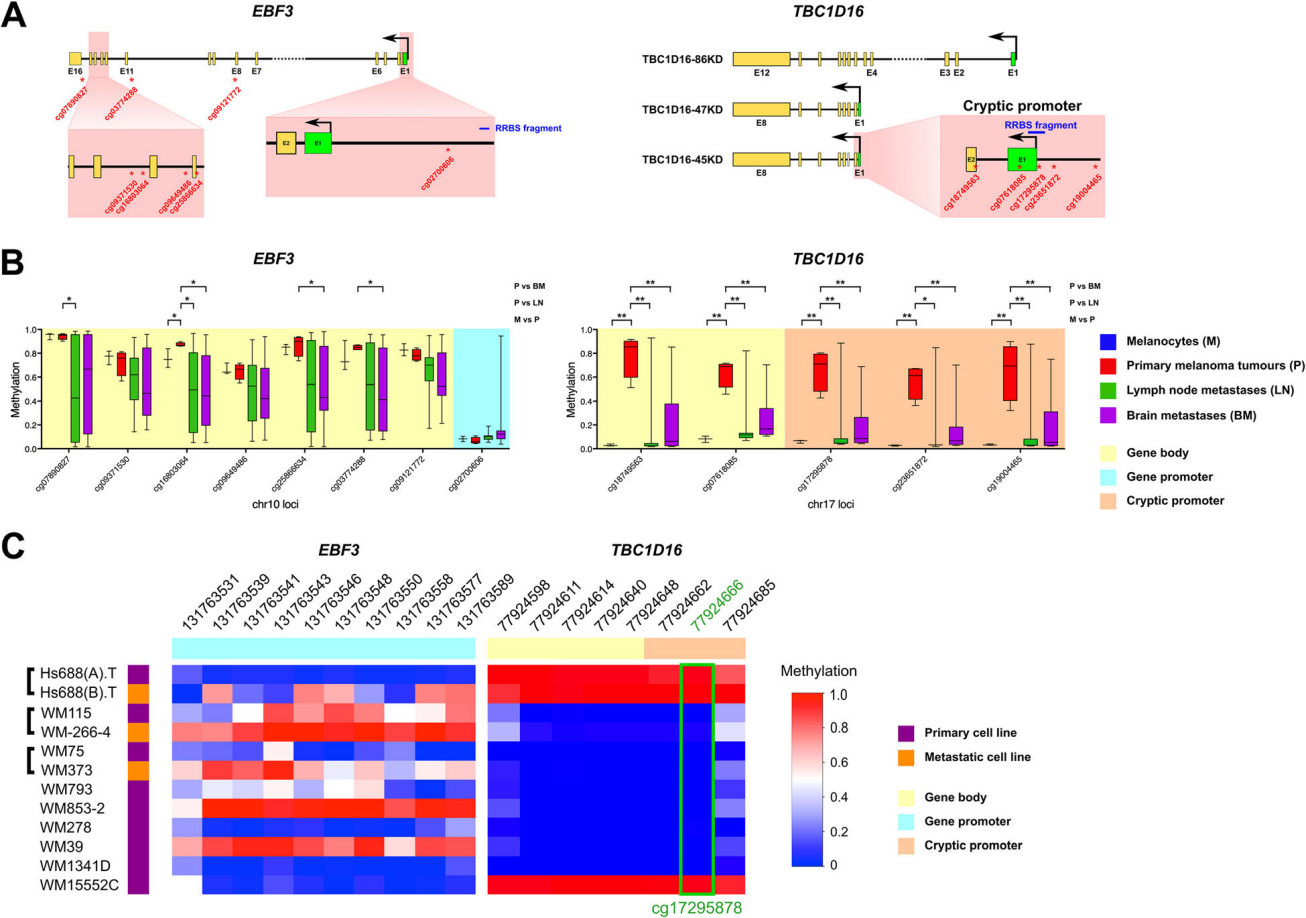
Analysis of *EBF3* and *TBC1D16* methylation in melanoma. **a** Gene maps for *EBF3* (left) and *TBC1D16* (right) show RRBS fragments (blue bars) and CpG sites (red asterisks) in relation to the transcription start site (TSS, black arrow), exon 1 (green box) and subsequent exons (gold boxes) of these genes. **b** Methylation analysis of *EBF3* and *TBC1D16* CpG sites in a melanoma dataset (accession number GSE44661 [12]) of normal melanocyte samples (M, *n* = 3, blue boxplots on the left), primary melanoma tumours (P, *n* = 4, red boxplots), lymph node metastases (LN, *n* = 17, green boxplots) and brain metastases (BM, *n* = 16, magenta boxplots); *P < 0.05, **P < 0.01. **c** DNA methylation patterns in *TBC1D16* from primary (purple) and matched metastatic (orange) melanoma cell lines derived from RRBS data. For *EBF3* promoter data from matched primary and metastatic melanoma cell line pairs, refer to Chatterjee et al. [9]. The cg17295878 site (marked with a green box) directly overlaps with a CpG site analysed in the melanoma tissue dataset above. Low to high methylation is shown as a continuous variable from blue (0) to white (0.5) to red (1)

We analysed CpG methylation in the promoter and gene body regions of *EBF3* and in the gene body of *TBC1D16*, which contains a promoter for the cryptic transcript *TBC1D16-47KD*, as summarised in the gene maps in Fig. 1a and listed in Table 1. Evaluation of *EBF3* CpG sites (at chr10:131636622–131671489, GRCh37; cg03774288, cg07890827, cg09121772, cg09371530, cg09649486, cg16803064 and cg25866634), in a cohort of 450k data of melanocytes (*n* = 3), primary melanomas (*n* = 4) and melanoma metastases (*n* = 33 in total) (Fig. 1b, left), showed that the *EBF3* gene body was hypomethylated (~ 40% loss of methylation on average) in melanoma metastases (green and magenta boxplots) compared to primary melanomas (red boxplots) or melanocytes (blue boxplots).

**Table 1:** Summary of analysed *EBF3* and *TBC1D16* CpG sites

Chromosome	GRCh37 loci	450k CpG site	RRBS coverage	Gene feature	Publication
chr10	131636623	cg07890827	NA	*EBF3*, gene body	[10]
	131640007	cg09371530			
	131640304	cg16803064			
	131641337	cg09649486			
	131641581	cg25866634			
	131647544	cg03774288			
	131671490	cg09121772			
	131763135	cg02700606		*EBF3*, promoter	[9]
	131763531	NA	58 bp fragment		
	131763539				
	131763541				
	131763543				
	131763546				
	131763548				
	131763550				
	131763558				
	131763577				
	131763589				
chr17	77924372	cg18749563	NA	*TBC1D16*, gene body	[10, 13]
	77924583	cg07618085			[13]
	77924598	NA	87 bp fragment		NA
	77924611				
	77924614				
	77924640				
	77924648				
	77924662			*TBC1D16-45/47KD*, cryptic promoter	
	77924666	cg17295878			[10, 13]
	77924685	NA			NA
	77924734	cg23651872	NA		[10]
	77925137	cg19004465			[13]

We also evaluated methylation of *TBC1D16* CpG sites (at chr17: 77924371–77925136, GRCh37; cg18749563, cg07618085, cg17295878, cg23651872 and cg19004465) in the same melanoma dataset (Fig. 1b, right). Several CpG sites in the gene body of *TBC1D16*, including the *TBC1D16-47KD* cryptic promoter, exhibited significant loss of methylation (− 47% on average) in metastatic melanomas (green and magenta boxplots) compared to primary tumours (red boxplots). In general, melanocytes were also hypomethylated in *TBC1D16*. We next evaluated *TBC1D16* methylation in an RRBS fragment that overlapped one of the CpG sites (cg17295878) in a series of metastatic melanoma cell lines (Fig. 1c). Consistent with the results from individual CpG sites, the matched and unmatched primary and metastatic melanoma cell lines exhibited hypomethylation of this RRBS fragment in most cases. No major differences were observed for methylation in *TBC1D16*, except in a primary and metastatic melanoma cell line pair and a primary melanoma cell line, which exhibited hypermethylation in both the *TBC1D16* gene body and *TBC1D16-47KD* cryptic promoter regions (Fig. 1c).

### DNA methylation differences in *EBF3* and *TBC1D16* between primary and metastatic tumours were identified in endometrial and prostate cancer tissues

**Fig. 2:**
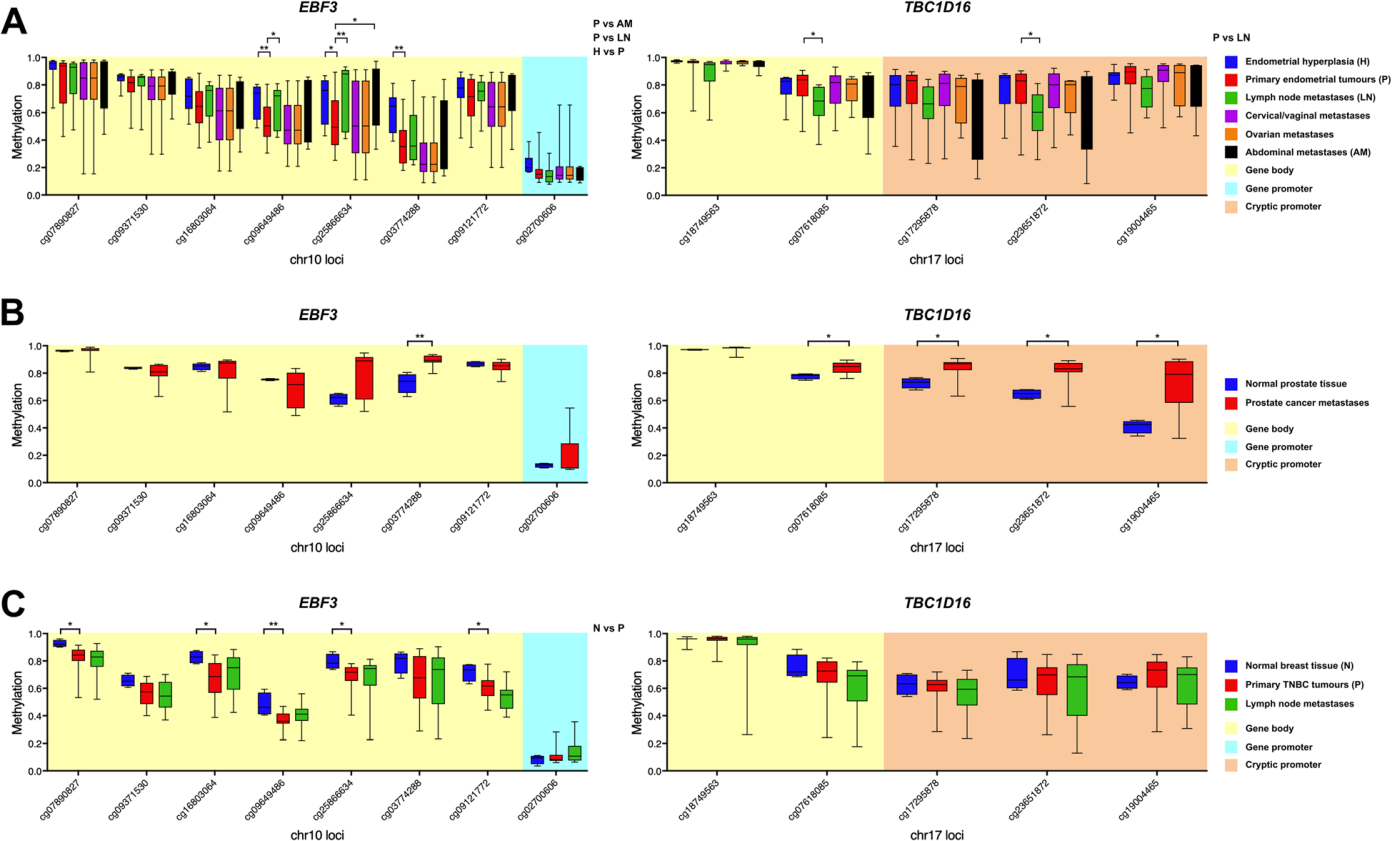
Analysis of *EBF3* and *TBC1D16* methylation in endometrial, prostate and breast cancers. Differentially methylated *EBF3* and *TBC1D16* CpG sites were analysed in Illumina 450k array DNA methylation data of **a** Teschendorff et al. dataset (accession number GSE67116 [14]) for endometrial hyperplasia (H, *n* = 8, in blue), primary endometrial tumours (P, *n* = 33, in red), lymph node metastases (LN, *n* = 11, in green), cervical/vaginal metastases (*n* = 26, magenta), ovarian metastases (*n* = 5, orange) and abdominal metastases (*n* = 8, black). **b** Aryee et al. dataset (accession number GSE38240[15]) for normal prostate (*n* = 4, blue) and prostate cancer metastases (*n* = 8, red). **c** Mathe et al. dataset (accession number GSE78758 [16]) for normal breast (N, *n* = 4, in blue), primary triple-negative breast cancer tumours (P, *n* = 23, in red) and lymph node metastases (*n* = 12, green); *P < 0.05, **P < 0.01

To investigate whether the findings from melanoma were generalisable to changes in methylation occurring during tumourigenesis or metastasis in multiple other cancer types, we next investigated previously published publicly available data from 450k methylation analysis of tissues of endometrial cancer (Fig. 2a), prostate cancer (Fig. 2b) and triple-negative breast cancer (Fig. 2c) to identify differences in methylation of *EBF3* and *TBC1D16* gene body and promoter regions.

In endometrial cancer (Fig. 2a), CpG sites in the gene bodies of *EBF3* (cg09649486, cg25866634) and *TBC1D16* (cg07618085) were significantly differentially methylated (+ 17% and − 14%, respectively) in lymph node metastases compared to primary tumours (green vs red boxplots). A similar significant loss of methylation was also observed in the *TBC1D16-47KD* cryptic promoter (cg23651872). One of the *EBF3* CpG sites (cg25866634) also gained methylation (+ 17%) in abdominal metastases (black vs red boxplots). Furthermore, three *EBF3* gene body CpG sites (cg09649486, cg25866634, cg03774288) showed a 19% gain of methylation in endometrial hyperplasia compared to primary endometrial tumours (blue vs red boxplots). In prostate cancer metastases (Fig. 2b) significant hypermethylation of one *EBF3* (cg03774288) gene body CpG and four *TBC1D16* (cg07618085, cg17295878, cg23651872, cg19004465) CpG sites (both + 16%) were observed compared to normal prostate tissue (red vs blue boxplots). For triple-negative breast cancer samples (Fig. 2c), there were no significant metastasis-related methylation changes in either the *EBF3* promoter or *TBC1D16*, but five CpG sites (cg07890827, cg16803064, cg09649486, cg25866634, cg09121772) in the *EBF3* gene body showed 12% loss of methylation in primary tumours compared to normal tissue (red vs blue boxplots).

### DNA methylation differences in *EBF3* and *TBC1D16* between primary and metastatic tumours were identified in colorectal cancer tissues

**Fig. 3:**
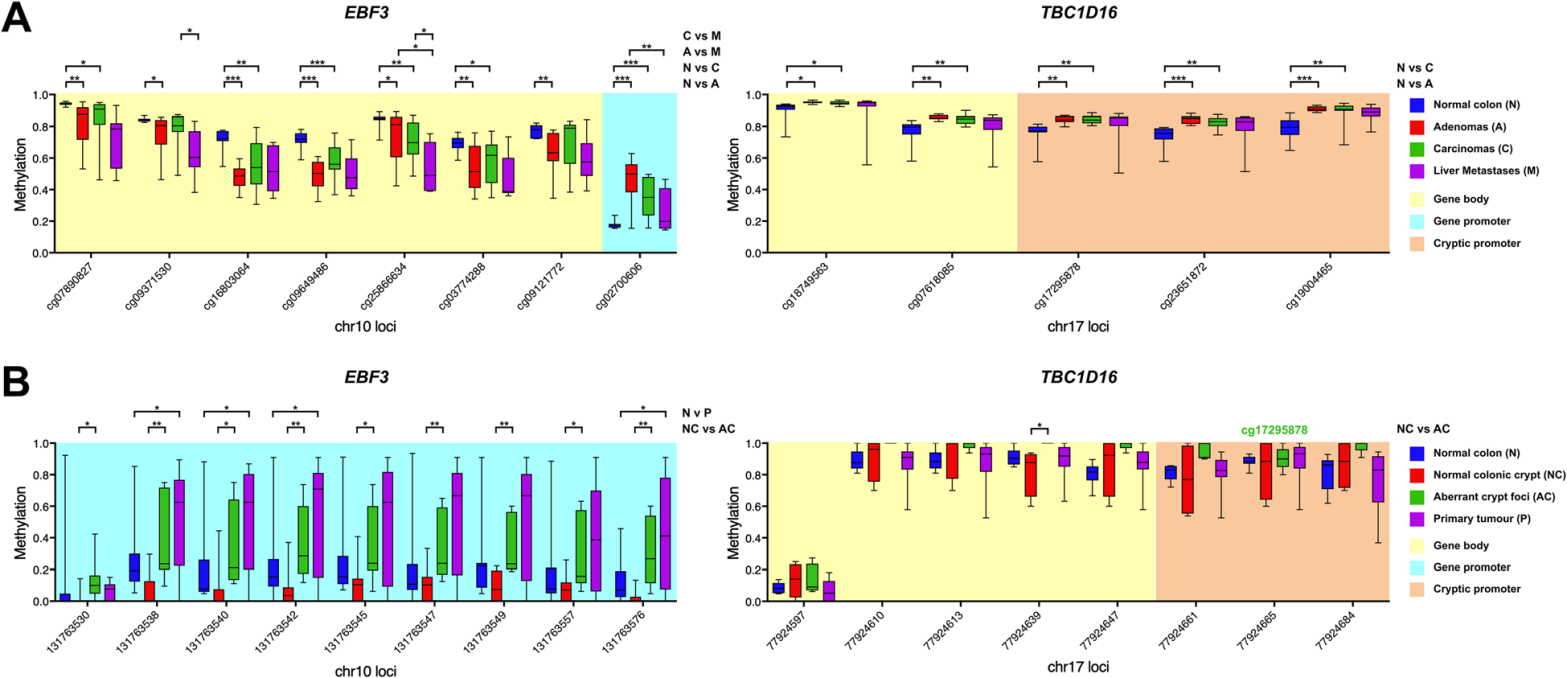
Analysis of *EBF3* and *TBC1D16* methylation in colorectal cancer. The differentially methylated *EBF3* and *TBC1D16* CpG sites were analysed in Illumina 450k array (**a**) and RRBS (**b**) DNA methylation datasets of colorectal cancer. **a** Qu et al. Illumina 450k array dataset (accession number GSE77954 [17]) for normal colon (N, *n* = 11, in blue), adenomas (A, *n* = 12, in red), carcinomas (C, *n* = 13, in green), and liver metastases (M, *n* = 9, in magenta). **b** The differentially methylated fragment in the *EBF3* promoter (chr10:131763530-131763587) identified by Chatterjee et al. [9] and the fragment in *TBC1D16* (chr17:77924597–77924683) directly overlapping the cg17295878 site (green) identified by Vizoso et al. [13] were analysed in an RRBS dataset (accession number GSE95654) of normal colon (N, *n* = 4, in blue), normal colonic crypt (NC, *n* = 8, in red), aberrant crypt foci (AC, *n* = 9, in green) and primary colorectal cancer tumour (P, *n* = 10, in magenta); *P < 0.05, **P < 0.01, ***P < 0.0001

We also evaluated methylation changes of *EBF3* and *TBC1D16* in 450k methylation array (Fig. 3a) and RRBS (Fig. 3b) datasets of colorectal cancer. Almost all analysed CpG sites in *EBF3* and *TBC1D16* were significantly differentially methylated (− 14% in the gene body of *EBF3* and + 9% in the gene body of *TBC1D16*) in both adenomas and carcinomas compared to normal colon tissue (red vs blue and green vs blue boxplots, respectively). In liver metastases, there was further 15% loss of methylation at several *EBF3* gene body sites compared to carcinomas (magenta vs green boxplots), and 19% loss of methylation compared to adenomas (magenta vs red boxplots, Fig. 3a). In the RRBS dataset, all *EBF3* promoter CpG sites and one *TBC1D16* site in the gene body were significantly hypermethylated (+ 26% and + 17%, respectively) in aberrant crypt foci compared to normal colonic crypt (green vs red boxplots). There were also four CpG sites in the *EBF3* promoter that were hypermethylated + 30% in primary tumours of the colon compared to normal colon (magenta vs blue boxplots, Fig. 3b).

### DNA methylation differences in *EBF3* and *TBC1D16* between normal and cancerous tissue types and cell lines were identified in whole genome bisulfite sequencing data

**Fig. 4:**
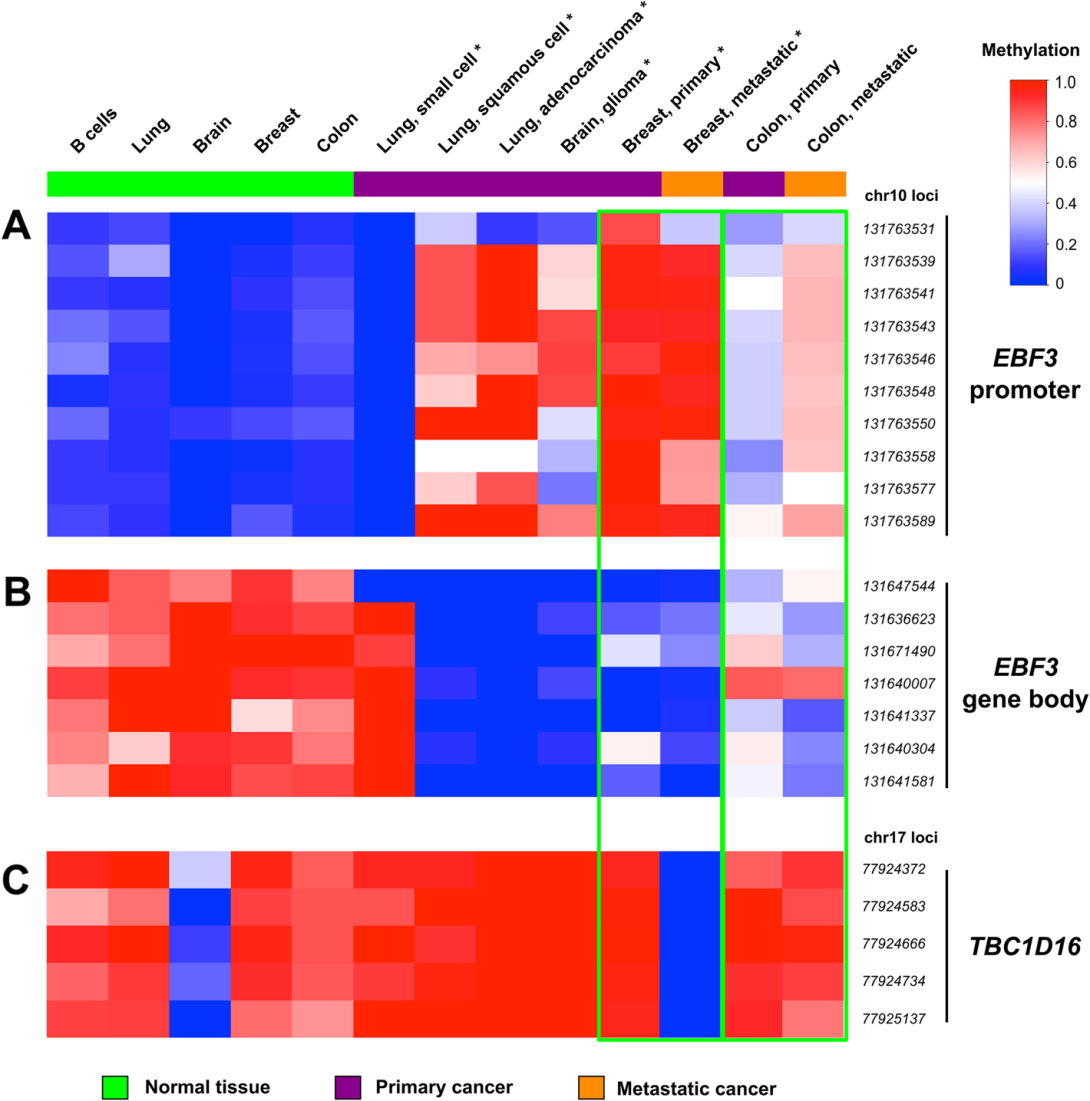
DNA methylation patterns of *EBF3* and *TBC1D16* CpG dinucleotides in a whole genome bisulfite sequencing (WGBS) cohort of normal tissues, primary tumours and metastases. These data (accession numbers GSE52271 and GSE56763 [18]) consist of normal tissues/cells (*n* = 5, green), primary tumours/cells (*n* = 6, purple) and metastases (*n* = 2, orange). Cell lines are denoted with an asterisk (*) and the primary and metastatic pairs are outlined with green boxes. **a** The upper panel shows ten CpG loci in the *EBF3* promoter identified as differentially methylated between primary and metastatic melanoma by Chatterjee et al. [9]. **b** The middle panel shows DNA methylation patterns of seven CpG sites in the gene body of *EBF3*, which were identified as differentially methylated between benign nevi, primary melanomas and metastases by Wouters et al. [10]. **c** The bottom panel shows a combination of five CpG sites in the gene body of *TBC1D16* that Vizoso et al. [13] and Wouters et al. [10] showed were differentially methylated between primary and metastatic tumours. Low to high methylation is shown as a continuous variable from blue (0) to white (0.5) to red (1)

To evaluate DNA methylation changes in both the promoter and gene body of *EBF3* and *TBC1D16*, we interrogated all analysed CpG sites in independent whole genome bisulfite sequencing (WGBS) data [18] derived from five different normal tissues/cell lines (B cells, lung, brain, breast and colon), six different primary tumours/cell lines (small cell lung carcinoma, squamous cell carcinoma of the lung, adenocarcinoma of the lung, glioma, primary breast cancer, primary colorectal cancer) and metastases (breast, colorectal) (Fig. 4). In all of the normal tissue samples, the *EBF3* promoter CpG sites were strikingly completely unmethylated (Fig. 4a, median = 0.08), and CpG sites in the gene body were mainly hypermethylated (Fig. 4b, median = 0.88). Interestingly, a small cell lung carcinoma cell line also showed this pattern. In the remaining primary cancer cell lines, the *EBF3* promoter was either hypermethylated or exhibited an increase in methylation compared to normal, whereas the gene body CpG sites were almost fully unmethylated. Overall, the *EBF3* promoter was hypermethylated in both primary and metastatic breast cancer and was unable to discriminate between them. In the metastatic colon cancer tumour sample, there was a clear increase in methylation (~ 20%) in the *EBF3* promoter compared to the primary colon cancer tumour sample, and a corresponding decrease in methylation (~ 20%) at most sites within the *EBF3* gene body in the metastatic vs primary colorectal cancer samples. With the exception of the normal brain tissue and the metastatic breast cancer cell line, *TBC1D16* was largely methylated (median = 0.94) in all of the cell lines and samples. In the breast cancer cell lines, *TBC1D16* methylation showed high discrimination between the primary (median = 0.96) and the metastatic (median = 0) breast cancer cell line pair (Fig. 4).

**Fig. 5:**
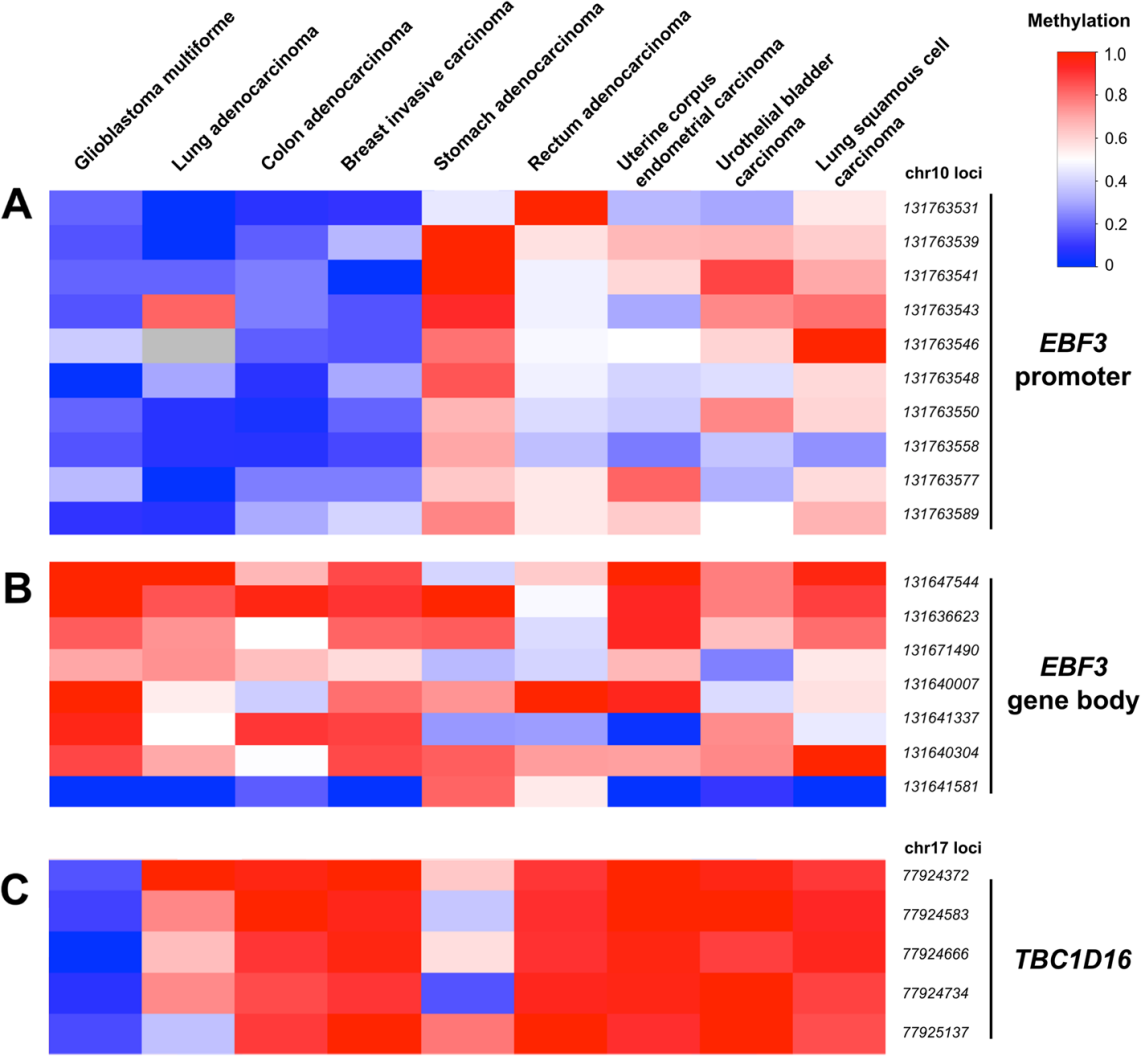
DNA methylation patterns of *EBF3* and *TBC1D16* CpG dinucleotides in a whole genome bisulfite sequencing (WGBS) cohort of primary tumours. These TCGA WGBS data consist of 46 primary tumour samples for urothelial bladder carcinoma (BLCA, *n* = 7), breast invasive carcinoma (BRCA, *n* = 5), colon adenocarcinoma (COAD, *n* = 3), glioblastoma multiforme (GBM, *n* = 6), lung adenocarcinoma (LUAD, *n* = 6), lung squamous cell carcinoma (LUSC, *n* = 5), rectum adenocarcinoma (READ, *n* = 3), stomach adenocarcinoma (STAD, *n* = 5) and uterine corpus endometrial carcinoma (UCEC, *n* = 6). **a** The upper panel shows ten CpG loci in the *EBF3* promoter identified as differentially methylated between primary and metastatic melanoma by Chatterjee et al. [9]. **b** The middle panel shows DNA methylation patterns of seven CpG sites in the gene body of *EBF3*, which were identified as differentially methylated between benign nevi, primary melanomas and metastases by Wouters et al. [10]. **c** The bottom panel shows a combination of five CpG sites in the gene body of *TBC1D16* that Vizoso et al. [13] and Wouters et al. [10] showed as differentially methylated between primary and metastatic tumours. Low to high methylation is shown as a continuous variable from blue (0) to white (0.5) to red (1)

To further assess the methylation of *EBF3* and *TBC1D16*, we also investigated the same CpG sites in additional WGBS of cancer samples from TCGA, consisting of primary tumour samples for urothelial bladder carcinoma, breast invasive carcinoma, colon adenocarcinoma, glioblastoma multiforme, lung adenocarcinoma, lung squamous cell carcinoma, rectum adenocarcinoma, stomach adenocarcinoma and uterine corpus endometrial carcinoma (Fig. 5). All of the *EBF3* promoter CpG sites were comparatively highly methylated in five tumour samples, stomach and rectum adenocarcinomas, uterine corpus endometrial carcinoma, urothelial bladder cancer and lung squamous cell carcinoma (Fig. 5a, median = 0.57), but comparatively hypomethylated in four tumour samples, glioblastoma multiforme, lung adenocarcinoma, colon adenocarcinoma and breast invasive carcinoma (median = 0.17). The *EBF3* gene body was comparatively highly methylated on average in all of the tumour samples (median = 0.75). *TBC1D16* methylation was high on average in all of the tumour samples (median = 0.86) except the glioblastoma multiforme sample (median = 0.14).

## Discussion

Here we have investigated CpG methylation alterations in the promoter and gene body of *EBF3* and the gene body of *TBC1D16*, which includes a cryptic promoter for *TBC1D16-47KD*. This investigation was performed on multiple tumour types in comparison to normal tissues, in matched metastatic vs primary tumour tissues or cell lines and in unmatched primary tumour and normal tissues and cell lines. Differences in methylation in the promoters and gene bodies were more pronounced in higher tumour grades or in metastatic tumour tissues and cell lines vs primary tumour tissues and cell lines.

Relatively greater hypomethylation in the *EBF3* gene body was observed in metastatic melanoma and colorectal cancer vs primary, in both tumour tissues and cell lines. Hypomethylation in the *EBF3* gene body was also observed in squamous cell carcinoma of the lung, adenocarcinoma of the lung, glioma and in colorectal cancer tissue or cell lines in comparison to normal colon tissues or cell lines. Conversely, *EBF3* promoter hypermethylation was also observed in metastatic or hyperplastic cancers vs primary cancers, in comparison to normal tissues of the aforementioned tumour and cell line types. Increased methylation of a CpG site in the *EBF3* promoter in primary and metastatic tumours (cg02700606, which is 396 bp from the RRBS fragment previously analysed [9] and additional sites examined) was consistent with previously published data showing significant hypermethylation of the *EBF3* promoter in metastatic melanoma cell lines [9]. We were unable to carry out a complete analysis of changes in CpG methylation in the *EBF3* promoter using 450k data, because there was no direct overlap of CpG sites in the *EBF3* promoter between RRBS and 450k array platforms. Nevertheless, evaluation of both *EBF3* promoter and gene body in WGBS data [18] (in Fig. 4) enabled us to examine the exact same CpG sites in normal and cancer tissues and cell lines, and the methylation changes were consistent with previously published data [9].

Of particular note, in a primary/metastatic pair of colorectal cancer samples, we found that the colorectal metastatic samples showed a ~ 20% increase in methylation in the *EBF3* promoter and a ~ 20% decrease in methylation at most sites in the gene body, which echoes previous observations made regarding the analysis of the *EBF3* promoter and the *EBF3* gene body in two independent studies of melanoma metastases [9, 10]. Taken altogether, these results suggest that the *EBF3* promoter hypermethylation and gene body hypomethylation is associated with tumour progression and metastasis, and it might be a characteristic aggressive change shared by both melanoma and colorectal cancer.

We also observed characteristic *TBC1D16* methylation changes in both melanoma and breast cancer (hypomethylation of the *TBC1D16* gene body, including the cryptic *TBC1D16-47KD* cryptic promoter (75%), in metastatic melanoma compared to primary melanoma and also hypomethylation (94%) in metastatic vs primary paired breast cancer samples). In both prostate and colorectal cancers, we observed hypermethylation of *TBC1D16* compared to normal prostate tissues and normal colon tissues, respectively. Hypomethylation of *TBC1D16* was previously reported in metastatic vs primary melanoma, breast cancer and other tumour types [10, 13]. However, no discernable methylation differences of *TBC1D16* were observed in a colorectal cancer primary/metastasis pair. Methylation differences in *TBC1D16* have previously been reported by Vizoso et al. [13] and were corroborated in findings by Wouters et al. [10]. Similar results were observed in this study, using the Marzese et al. cohort [11, 12] and in the WGBS data [18], which support the notion that methylation changes in *TBC1D16* are a potential epigenetic driver of tumour metastasis.

However, epigenetic changes in *EBF3*, or *TBC1D16*, were not identified in all tumour samples, or in all primary/metastasis cancer pairs. For example, no clear overall pattern of *EBF3* methylation patterns was identified between a paired breast cancer primary and metastases, although some individual sites showed differences (e.g. the CpG site at chr10:131763531 was 46% less methylated in metastasis). In addition, the direction of methylation changes in *EBF3* and *TBC1D16* was not necessarily conserved across different cancer types, since endometrial cancer and prostate cancer in our data showed opposite patterns of methylation changes in *EBF3* gene body and promoter regions compared to melanoma and colorectal cancer.

We show here that in several cancer types, characteristic methylation changes in both *EBF3* and *TBC1D16* were associated with tumour metastasis, which is responsible for the majority of cancer-related deaths. As a first step of metastasis, tumour cells are released from a solid tumour and circulate in the bloodstream of patients. Molecular signatures of these circulating tumour cells (CTCs), such as the methylation status of *EBF3* and *TBC1D16*, could potentially be used as a biomarker to help determine the prognosis of metastatic cancers. Furthermore, if the identified methylation changes are causal, they may have significant relevance to early diagnosis of cancer and possibly as a therapeutic target. For example, it appears that the *EBF3* promoter hypermethylation identified by RRBS [9] may potentially be an early universal marker for detecting the presence of several cancer types, which have undergone aberrant methylation. As this region is completely unmethylated across many normal somatic tissue types and gains methylation in different stages of cancer, it would be relatively easy to detect.

We acknowledge that a full interpretation of the methylation results presented here should take into consideration different analysis platforms, each having their own strengths and weaknesses. For instance, in contrast to WGBS, where all CpGs in the genome were analysed, RRBS enriched for CpG-dense regions and involved sequencing of four million CpG sites but only 13.4% of the genome [19]. On the other hand, the Illumina 450k platform used by Vizoso et al. [13], Marzese et al. [11, 12] and Wouters et al. [10] could enable genome-wide methylation profiling [20], but the majority of the probes in this platform were located around gene promoters and CpG islands, whereas methylation changes in the cancer epigenome were often observed at higher levels in other genomic segments such as gene bodies and intergenic regions [21–24]. Furthermore, single CpG sites are more likely to yield stochastic variation in methylation profiles [25]. The importance of taking into consideration the different analysis platforms used [26] is highlighted by the fact that the *EBF3* promoter hypermethylation identified by RRBS [9] was not able to be identified by 450k, regardless of sample numbers, due to the absence of probes for this region. Likewise, the *TBC1D16* CpG sites identified by 450k were not detected by RRBS due to the lack of MspI fragments encompassing the majority of those specific loci. Further sequencing-based studies, such as WGBS, will provide a more comprehensive view of the cancer methylome and will help to identify greater numbers of epigenetic markers that distinguish between primary and metastatic tumour pairs.

## Conclusions

The present findings suggest that methylation changes in *EBF3* and *TBC1D16*, similar to those reported previously [9, 10], are found in multiple different tumour types and are associated with aggressive tumour behaviour. Overall, the findings presented strengthen the view that methylation changes in *EBF3* and *TBC1D16* are potential epi-drivers of aggressive tumourigenic changes in multiple cancer types.

## Methods

### Analysis of Illumina 450k array DNA methylation data in independent cancer cohorts

All 450k methylation data were obtained from the NCBI gene expression omnibus (GEO) database (URL https://www.ncbi.nlm.nih.gov/geo/): (1) 16 melanoma metastases to the brain, 17 lymph node melanoma metastases, 4 primary melanoma tumours and 3 normal melanocyte samples (accession number GSE44661[12], data retrieved on 11/6/2015); (2) 8 endometrial hyperplasia, 33 primary endometrial tumours, 11 lymph node metastases, 26 cervical/vaginal metastases, 5 ovarian metastases and 8 abdominal metastases (accession number GSE67116[14], data retrieved on 2/3/2018); (3) 4 normal prostate tissues and 8 prostate cancer metastases (accession number GSE38240[15], data retrieved on 5/3/2018); (4) 4 normal breast tissues, 23 primary triple-negative breast cancer tumours and 12 lymph node metastases (accession number GSE78758[16], data retrieved on 5/3/2018); (5) 11 normal colon tissues, 12 adenomas, 13 carcinomas and 9 liver metastases (accession number GSE77954[17], data retrieved on 9/7/2018).

### Analysis of RRBS DNA methylation data in an independent colorectal cancer cohort

An RRBS dataset was obtained from the GEO database (accession number GSE95654, data retrieved on 10/7/2018) of four normal colon, eight normal colonic crypt, nine9 aberrant crypt foci and ten primary colorectal cancer tumours.

### RRBS data analysis of melanoma cell lines

Reduced representation bisulfite sequencing (RRBS) libraries for the 12 cell lines described here (in Fig. 1c) were prepared according to previously published protocols [19, 27, 28]. The cell lines analysed were Hs688(A).T (primary melanoma) and Hs688(B).T (matching metastatic melanoma), WM75 (primary melanoma) and WM373 (matching metastatic melanoma), WM115 (primary melanoma) and WM-266-4 (matching metastatic melanoma) and six additional primary melanoma cell lines, WM793, WM853-2, WM278, WM39, WM1341D and WM15552C. Quality assessment, mapping of the sequencing reads and extraction of CpG methylation data were performed using the DMAP pipeline [29].

### Analysis of WGBS DNA methylation data in independent cancer cohorts

(1) WGBS data for five normal tissues/cells (CD19+ B cells, lung tissue, brain white matter, breast tissue and colon tissue), six primary tumours/cells (H1672 small cell lung carcinoma cells, H157 squamous cell carcinoma of the lung cells, H1437 adenocarcinoma of the lung cells, U87MG glioma cells, 468PT breast cancer cells and colorectal cancer tissue) and two metastases (468LN breast cancer cells, colorectal cancer tissue) were obtained from GEO (accession numbers GSE52271 and GSE56763 [18], data retrieved on 23/06/2017).

(2) TCGA WGBS data for seven urothelial bladder carcinoma (TCGA-BLCA), five breast invasive carcinoma (TCGA-BRCA), three colon adenocarcinoma (TCGA-COAD), six glioblastoma multiforme (TCGA-GBM), six lung adenocarcinoma (TCGA-LUAD), five lung squamous cell carcinoma (TCGA-LUSC), three rectum adenocarcinoma (TCGA-READ), five stomach adenocarcinoma (TCGA-STAD) and six uterine corpus endometrial carcinoma (TCGA-UCEC) were obtained from http://zwdzwd.io/trackHubs/TCGA_WGBS/hg38/bw_mindepth5/ (data retrieved on 30/7/2018). The bigWig files were converted to BedGraph format using bigWigToBedGraph (http://hgdownload.cse.ucsc.edu/admin/exe/linux.x86_64/).

### Data collation and statistical analysis

The BEDTools suite (https://bedtools.readthedocs.io/) was used to extract methylation beta values for all analysed loci. Datasets were collated and non-parametric Mann-Whitney *U* statistical tests were performed in the R Studio environment (version 3.1.1).

## Data Availability

The 450k (GSE44661, GSE67116, GSE38240, GSE78758, GSE77954), RRBS (GSE95654, GSE70621) and WGBS (GSE52271, GSE56763) methylation datasets analysed during the current study are available in the NCBI GEO repository, https://www.ncbi.nlm.nih.gov/geo/. The additional RRBS data generated during the current study (primary melanoma cell lines WM793, WM853-2, WM278, WM39, WM1341D and WM15552C) are available from the corresponding author on reasonable request.

## Abbreviations

BLCA: Urothelial bladder carcinoma
BRCA: Breast invasive carcinoma
COAD: Colon adenocarcinoma
CTC: Circulating tumour cell
EBF3: Early B Cell Factor 3
GBM: Glioblastoma multiforme
GEO: Gene expression omnibus
LUAD: Lung adenocarcinoma
LUSC: Lung squamous cell carcinoma
READ: Rectum adenocarcinoma
RRBS: Reduced representation bisulfite sequencing
STAD: Stomach adenocarcinoma
TBC1D16: TBC1 domain family member 16
TCGA: The Cancer Genome Atlas
UCEC: Uterine corpus endometrial carcinoma
WGBS: Whole genome bisulfite sequencing
